# Analysis of immune responses to attenuated alcelaphine herpesvirus 1 formulated with and without adjuvant

**DOI:** 10.1016/j.jvacx.2021.100090

**Published:** 2021-03-22

**Authors:** George C. Russell, David M. Haig, Mark P. Dagleish, Helen Todd, Ann Percival, Dawn M. Grant, Jackie Thomson, Anna E. Karagianni, Julio Benavides

**Affiliations:** a Moredun Research Institute, Pentlands Science Park, Midlothian EH26 0PZ, UK; b School of Veterinary Medicine and Science, University of Nottingham, Sutton Bonington Campus, Leicestershire LE12 5RD, UK

**Keywords:** Malignant catarrhal fever, Vaccine, Emulsigen, Inactivation, Antigen, Antibody

## Abstract

The experimental vaccine for bovine malignant catarrhal fever consists of viable attenuated alcelaphine herpesvirus 1 (AlHV-1) derived by extensive culture passage, combined with an oil-in-water adjuvant, delivered intramuscularly. This immunisation strategy was over 80% effective in previous experimental and field trials and protection appeared to be associated with induction of virus-neutralising antibodies. Whether the vaccine virus is required to be viable at the point of immunisation and whether adjuvant is required to induce the appropriate immune responses remains unclear. To address these issues two studies were performed, firstly to analyse immune responses in the presence and absence of adjuvant and secondly, to investigate immune responses to vaccines containing adjuvant plus viable or inactivated AlHV-1.

The first study showed that viable attenuated AlHV-1 in the absence of adjuvant induced virus-specific antibodies but the titres of virus-neutralising antibodies were significantly lower than those induced by vaccine containing viable virus and adjuvant, suggesting adjuvant was required for optimal responses. In contrast, the second study found that the vaccine containing inactivated (>99.9%) AlHV-1 induced similar levels of virus-neutralising antibody to the equivalent formulation containing viable AlHV-1.

Together these studies suggest that the MCF vaccine acts as an antigen depot for induction of immune responses, requiring adjuvant and a suitable antigen source, which need not be viable virus. These observations may help in directing the development of alternative MCF vaccine formulations for distribution in the absence of an extensive cold chain.

## Highlights

• MCF vaccine was tested with and without adjuvant and containing inactivated virus.
• Adjuvant was required for optimal virus neutralising antibody responses.
• Storage of AlHV-1 with Emulsigen adjuvant significantly reduced virus viability.
• Vaccination with adjuvant-inactivated AlHV-1 did not reduce antibody responses.

## 1 Introduction

Malignant catarrhal fever (MCF) is a usually-fatal lymphoproliferative disease affecting a range of ungulates caused by members of the Macavirus genus of gammaherpesviruses, particularly alcelaphine herpesvirus 1 (AlHV-1) and ovine herpesvirus 2 (OvHV-2) [1], [2], [3]. Animals that exhibit clinical signs of MCF seldom survive, although there is evidence that sub-clinical infection may occur [4], [5]. AlHV-1 is carried asymptomatically by wildebeest but can be transmitted to disease-susceptible ungulates causing significant losses in areas of eastern and southern Africa where wildebeest and livestock come into contact and also in zoological collections worldwide [1], [2]. MCF does not appear to transmit horizontally between affected animals, suggesting clinical disease occurs only in dead-end hosts, but examples of vertical transmission in MCF reservoir and susceptible species have been recorded [6], [7], [8]. OvHV-2 is carried asymptomatically by sheep and sheep-associated (SA-) MCF is a problem worldwide wherever reservoir and disease-susceptible hosts cohabit. Globally, MCF has both economic and animal welfare impacts [2], [9], [10], [11], [12].

Alcelaphine herpesviruses 1 and 2 are the only Macaviruses that can currently be propagated in culture [13], [14], although AlHV-2 was considered non-pathogenic in cattle, leading to the preferential use of AlHV-1 over OvHV-2 to study the pathogenesis and control of MCF [15], [16], [17], [18], [19], [20], [21]. Pathogenic AlHV-1 can be propagated in a cell-associated form in bovine primary epithelial cells but after several passages the virus becomes culture-adapted and releases cell-free virus into the culture medium [22], [23]. This culture adaptation is associated with loss of virulence, such that the resultant virus is unable to cause MCF when used to infect cattle and rabbits [23], [24]. The loss of virus pathogenicity is associated with genomic changes, including rearrangements and deletions [24, Russell, unpublished data] and has allowed the development of a vaccine against AlHV-1 MCF [19]. The high passage AlHV-1 C500 virus is a stable attenuated virus strain which, when formulated with Freunds’ complete and incomplete adjuvants in an intramuscular two-dose strategy, protected cattle from an intranasal challenge with the pathogenic C500 parent strain of AlHV-1 that was usually fatal in unvaccinated animals [19]. This live-attenuated vaccine, using the licensed oil-in-water adjuvant Emulsigen (https://mvpadjuvants.com/wp-content/uploads/2017/10/PHIB-17018-Adjuvants-Bulletin_Emulsigen.pdf), was found to be safe and effective in experimental and field trials involving hundreds of cattle [4], [20], [21], [25], [26].

However, it remains unclear whether this vaccine requires the presence (and replication) of viable virus to function optimally or if it simply provides a source of virus antigen. Here we report the results of experiments in which the attenuated AlHV-1 vaccine strain was used to immunise cattle in the absence of adjuvant, mixed with adjuvant at the point of immunisation or stored in adjuvant until virus viability was reduced by over 99.9%. We show that the attenuated AlHV-1 MCF vaccine requires an adjuvant to induce optimal (virus neutralising) immune responses but the virus need not be viable. These results are an important advance in our characterisation of the vaccine formulation to allow its uptake commercially, especially in the absence of a cold chain.

## 2 Materials and methods

### 2.1 Animals

Groups of 6–8 disease-free and AlHV-1 seronegative male Ayrshire, Holstein or Friesian-Holstein cross *Bos taurus* calves of 3–7 months of age were used in the experiments. All animals recruited were housed at the Moredun Research Institute animal accommodation for at least one week before the start of any procedures to allow their health status to be evaluated and to allow acclimatisation. Power calculations based on an estimated frequency of disease of 80% in unvaccinated cattle and 10% in vaccinated cattle after experimental challenge; a false positive rate of 0.05; and power of 80% were used to derive the group sizes used. All animal experiments were performed with the approval of the Moredun Research Institute’s Animal Welfare and Ethical Review Body (AWERB) in compliance with the UK “Animals (Scientific Procedures) Act 1986”, which included a statistical review of group sizes. The experimental design submitted for ethical review included a statement that animals would be monitored daily for health and well-being and that any animal developing more than mild clinical signs would be euthanased for welfare reasons. One animal was withdrawn from study 2 (group 2A) before the second immunisation due to respiratory disease; the data from all other animals were included in the analysis.

Individual units - animals - were assigned to experimental groups randomly, while ensuring an even distribution of animal ages and source locations as follows: animals were sorted by birth location and UK animal number and then were assigned to groups A, B and C in order. The groups in each study had average ages that differed by less than one week. Staff involved in animal care were blinded to the group allocations at all stages in the studies, while the scientists involved in sample/data analysis were not. No subjective judgements were involved in the analysis of samples.

### 2.2 Immunisation study design

Table 1 shows the details of the two vaccine studies performed. In study 1, three groups of at least six calves were immunised with: attenuated AlHV-1 mixed with 20% Emulsigen immediately before use (group 1A, n = 6); attenuated AlHV-1 without adjuvant (group 1B, n = 6); and culture medium only (group 1C, n = 7). Virus titre in the inoculum was 10^6^–10^7^ 50% tissue-culture-infectious dose (TCID50)/ml. Animals were given two doses of the same vaccine formulation as intramuscular (upper neck) inoculations on day 0 (d0) and d28 and were sampled for blood and nasal secretions on d0, d14, d28, d47, d56 and d69. Blood plasma and nasal secretions (NS) were analysed for virus-specific antibodies by ELISA, while d0 and d69 samples were tested for virus-neutralising antibodies, to qualitatively evaluate the antibody responses.

**Table 1:** Details of vaccine studies.

Study	*Group (n)	$Prime (d0)	Boost (d28)	†Sampling days (d)	‡assays
**1**	1A (6)	Viable AlHV-1 /20% Emulsigen	Viable AlHV-1 /20% Emulsigen	Blood and NS: d0, d14, d28, d47, d56, d69	ELISA, VNA
	1B (6)	Viable AlHV-1	Viable AlHV-1		
	1C (7)	Medium only	Medium only		
**2**	2A (7)	Viable AlHV-1 /20% Emulsigen	Viable AlHV-1 /20% Emulsigen	Blood and NS: d0, d28, d56	ELISA, VNA
	2B (8)	Inactivated AlHV-1 /20% Emulsigen	Inactivated AlHV-1 /20% Emulsigen		
	2C (8)	20% Emulsigen	20% Emulsigen		

* Group designations and numbers of animals per group (n). One animal was withdrawn from study 2 (group 2A) before boost due to respiratory disease.

$ Immunisations (each 1 mL, given intramuscularly in the upper neck as prime on day 0 (d0), boost on d28).

† Sampling days (d) are expressed as days after first immunisation; sample types as Blood (uncoagulated blood) and NS (nasal secretions).

‡ Assays performed are shown as ELISA (AlHV-1 ELISA) and VNA (AlHV-1 virus neutralisation assay).

In study 2 (Table 1) three groups of 8 calves were immunised with: attenuated AlHV-1 vaccine containing 20% Emulsigen (~10^7^ TCID50/ml), formulated on the day of immunisation (group 2A); identically constituted vaccine that had been stored at 4 °C with mixing for four weeks and whose AlHV-1 viable titre (~10^3^ TCID50/ml at the point of vaccination) had been reduced by more than 99.9% (group 2B); and medium containing 20% Emulsigen (group 2C). As in study 1, the animals in each group received two doses of the same vaccine formulation as intramuscular (upper neck) inoculations on d0 and d28. One animal in group 2A was euthanased for welfare reasons before the second immunisation. Samples of blood and nasal secretions were taken on d0, d28 and d56, and was concluded on day 56, four weeks after booster vaccination, while the virus-specific antibody response remained high (Table 1). Antibody responses were analysed by AlHV-1 specific ELISA and by virus neutralisation assay (VNA), as for study 1, focusing on a time point representing the peak of immune response (d56).

### 2.3 Cells, viruses and adjuvants

The strains of AlHV-1 used for vaccination and challenge were as described previously [19], [25]. Briefly, the attenuated AlHV-1 C500 strain at passage >1000 was used as the source of virus for immunisation. This cell-free virus was propagated in primary bovine turbinate (BT) cells at low passage and virus-containing culture supernatants were clarified by centrifugation and stored at −80 °C. Representative aliquots of attenuated AlHV-1 were titrated by serial dilution on BT cells as described previously [19] and are expressed as TCID50/ml.

Emulsigen (MVP Adjuvants, Phibro Animal Health, USA) is a licensed oil-in-water adjuvant that has no ingredients of animal origin. It contains micron-sized oil droplets with a high surface area available for antigen coating and does not cause adverse reactions at the injection site. Previous analysis of the attenuated AlHV-1 virus in combination with this adjuvant showed that 20% Emulsigen did not significantly reduce the titre of the virus in the 24 h after mixing and that the adjuvant was not toxic to BT cells [25] in virus titrations.

### 2.4 Virus viability assays

Aliquots of a single stock of attenuated AlHV-1 C500 virus were incubated at 4 °C or 20 °C in the presence or absence of 20% Emulsigen for periods of up to two months. Samples were mixed daily by inversion. After incubation, the proportion of viable AlHV-1 remaining in each sample was assayed by virus titration (TCID50) on BT cells by serial dilution as described [19]. The setting up of each replicate set was organised to allow the virus titrations to be performed together, enhancing the reliability of the assays.

### 2.5 Detection of AlHV-1 DNA

The presence of AlHV-1 DNA in immunised animals was assayed in purified genomic DNA samples extracted from peripheral blood buffy coat cells by real-time PCR as described previously [25], [27]. Briefly, 50–100 ng of total DNA was assayed by duplex (2-colour) real-time PCR analysis that simultaneously assayed for the presence of AlHV-1 DNA and bovine genomic β-actin specific DNA sequences (as control for the extraction and PCR processes) on an ABI 7000 or 7500 sequence detection system (Applied Biosystems).

### 2.6 Analysis of antibody responses to AlHV-1 by ELISA and virus neutralising antibody test

To quantify the humoral antibody response in blood plasma and the mucosal antibody response in nasal secretions (NS), an antibody ELISA, based on a detergent extract of attenuated AlHV-1, was used as described previously [25]. Briefly, pairs of adjacent rows of 96-well microtitre plates (Greiner, high protein binding) were coated with 50 μL of 5 μg/mL virus-positive or virus-negative antigen in 0.1 M carbonate buffer, pH 9.6. Individual samples of blood plasma or sterile-filtered nasal secretion fluid diluted in PBS were then applied in duplicate to positive and negative antigen wells at a dilution optimised in previous experiments (1/200 for plasma, 1/50 for NS). A similar dilution of an AlHV-1-positive plasma or NS pool was included on each plate as a positive control and to ensure reproducibility between assays. Known negative samples of plasma and NS were included with each test sample series. Antibody bound in each well was detected using 1:1000 dilution of rabbit anti-bovine IgG-Horseradish Peroxidase conjugate (Sigma). The plates were then washed and Sureblue TMB-peroxidase substrate (KPL) applied for five minutes. The reaction was stopped by the addition of 0.1 M HCl and the plates were read at 450 nm in a plate reader. ELISA values (difference between means of positive and negative antigen wells for each sample dilution) were normalised between plates by calculation of the sample-to-positive (s/p) ratio for each test sample, based on the positive control samples included on each plate, as described previously [25].

The virus neutralisation assay (VNA) was based upon inhibition of AlHV-1-induced cytopathic effect in BT cells by AlHV-1 virus neutralising antibodies in dilutions of plasma or nasal secretion fluid as described previously [19]. Assays were performed in 96 well tissue culture plates with BT cells at greater than 80% confluence. All assays used a high titre bovine anti-AlHV-1 serum as a positive control and included non-specific toxicity control wells containing sample and cells without virus. VNA titres were expressed as <2 when no neutralisation was detected at the lowest dilution that could be used (1:2 final).

### 2.7 Statistical analysis

After testing for normality, comparison of immune response data was performed by *T*-test on log_2_-transformed VNA titre data or directly on ELISA s/p ratios within Microsoft Excel. Statistical significance was assumed at p < 0.05. Immune response data were also subjected to the non-parametric Mann-Whitney *U* test using an online calculator (www.socscistatistics.com/tests/mannwhitney/), which allowed data to be analysed without transformation.

## 3 Results

### 3.1 Analysis of AlHV-1 viability in the presence and absence of adjuvant

**Fig. 1:**
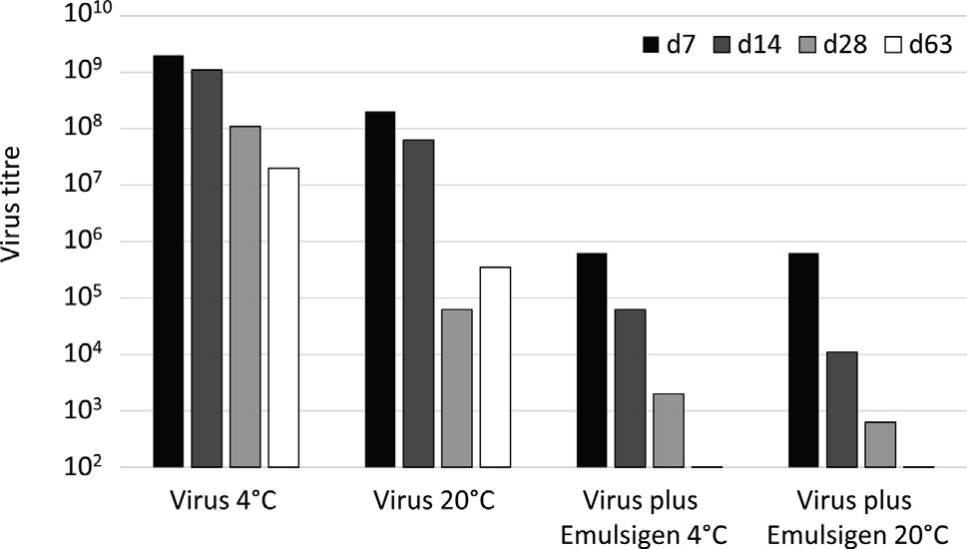
Chart of AlHV-1 viability when stored in the absence or presence of Emulsigen (20%) at 4 °C or 20 °C. Conditions of storage (virus or virus plus Emulsigen) are indicated below each group of columns. Storage times were as follows: 7 days, black columns; 14 days, dark grey columns; 28 days, light grey columns; 63 days, white columns. Values below the detection limit of the assay (10^2^ TCID50/ml) are shown at that level.

Titration of attenuated AlHV-1 after combination with Emulsigen in previous vaccine studies showed that mixing with adjuvant led to a less than ten-fold reduction in titre during the first 24 h and that 20% Emulsigen in culture medium was not toxic to BT cells in the context of virus titration assays [25]. In order to study longer-term vaccine virus stability when formulated with adjuvant, aliquots of attenuated AlHV-1 were incubated in the presence or absence of Emulsigen (20%) with daily mixing for up to two months. The resulting titre data are summarised in Fig. 1 and presented in Supplementary Data, Table 1.

In the absence of adjuvant, storage at 4 °C led to an approximately ten-fold loss in titre after one month, and a hundred-fold reduction after two months. Incubation at 20 °C led to a more rapid loss of viability, with reductions of about 10^3^-fold observed at both one and two months. Inclusion of Emulsigen caused a rapid and progressive loss of titre at both 4 °C and 20 °C, with virus titre reduced to approximately 10^3^ TCID50/ml after one month and to undetectable levels (<10^2^ TCID50/ml) after two months (Fig. 1).

### 3.2 Study 1: immune response to attenuated AlHV-1 in the presence and absence of adjuvant

**Fig. 2:**
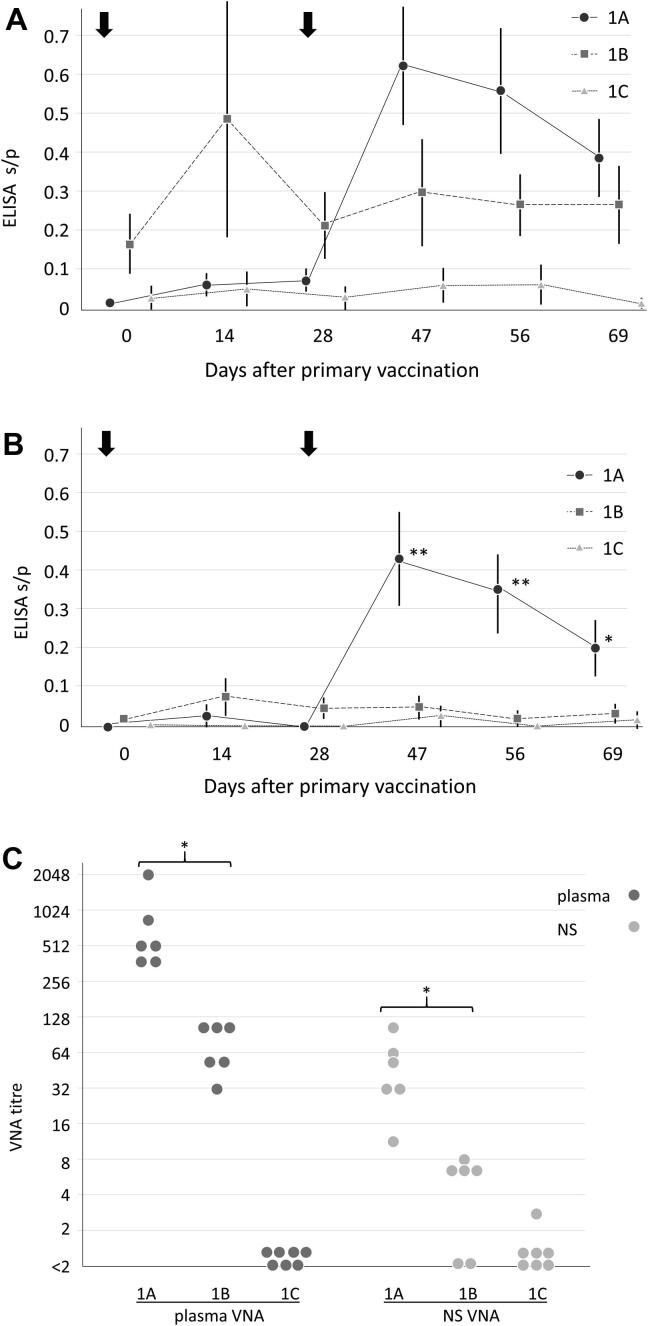
Study 1; ELISA and VNA data. In panels A and B the mean s/p value for each group was plotted against day of study for the respective samples as follows: group 1A (viable AlHV-1 mixed with 20% Emulsigen on the day of immunisation), dark grey circles and solid lines; group 1B (viable AlHV-1 only), mid grey squares and dashed lines; and group 1C (culture medium only), light grey triangles and dotted lines. Variation within each group is indicated by vertical black bars representing the sample standard deviation. Prime immunisation was on d0, boost on d28 (arrows). **(A)** Plasma ELISA time course; **(B)** nasal secretion (NS) ELISA time course. *T*-test comparison of NS s/p values in group 1A and group 1B showed significant differences at d47, d56 (**, p < 0.01) and at d69 (*, p < 0.05). **(C)** Virus-neutralising assay (VNA) data. The d69 VNA titres assayed in blood plasma (plasma VNA, dark grey circles) and nasal secretions (NS VNA, light grey circles) were plotted on a log2 scale by sample type and immunisation group as indicated beneath the figure. Samples with identical values in the same group were plotted offset to each other. Differences between groups 1A and 1B were considered significant (* p < 0.0005, *T*-test; p < 0.01, Mann-Whitney *U* test).

To determine whether the MCF vaccine required adjuvant for optimal performance, the immune response induced by immunisation with attenuated AlHV-1 with and without Emulsigen was tested in study 1. Plasma ELISA showed that AlHV-1-specific antibodies were induced by both the adjuvant-containing (group 1A) and adjuvant-free (group 1B) immunisations (Fig. 2A), while the control group (1C) had no such response. In contrast, only group 1A developed NS ELISA s/p values that were significantly higher than the control group (1C) (Fig. 2B). In the adjuvant-vaccine group (1A), the virus-specific antibody response remained low (median and range <0.1) until after the booster immunisation at d28 and peaked about 4 weeks later when s/p values rose to medians of 0.4–0.6 in plasma (range 0.6–1.0) and 0.2–0.4 in NS (range 0.4–0.6) on d47, d56 and d69 (Fig. 2A, B). In contrast, the group immunised with viable virus without adjuvant (1B) showed intermediate plasma s/p values (medians of 0.15–0.30; ranges 0.41–1.57) at all time points in the study, with one animal in this group having higher s/p values (1.7 plasma; 0.27 NS) at d14 than the rest of group 1B (Supplementary Data, Table 2). In this group, there also appeared to be no clear response to the booster immunisation at d28 (Fig. 2A, B). By T-test, group 1B values were not significantly different from the group 1A values at any timepoint in the plasma ELISA dataset (Fig. 2A; p > 0.08), but NS ELISA s/p values were significantly higher in group 1A at d47, d56 (p < 0.01) and d69 (p < 0.05; Fig. 2B).

Analysis of the VNA titres for both plasma and NS at d0 showed no background of AlHV-1 neutralising activity, as expected, while d69 samples showed a similar pattern to the ELISA data, with the lowest values in group 1C (only one animal had a detectable VNA titre of 3), intermediate values in group 1B (plasma median titre 68, range 32–90; NS median 6, range <2–8) and highest values in group 1A (plasma median 512, range 360–2048; NS median 39, range 11–90; Fig. 2C). The group 1B VNA titres were significantly lower than group 1A for both plasma and NS, with about ten-fold lower geometric mean titre (p < 0.0005, *T*-test; p < 0.01, Mann-Whitney *U* test).

Real-time PCR analysis, of DNA samples prepared from blood buffy coat cells, showed no detection of AlHV-1 DNA in the circulation of immunised animals at any time point during this trial, while genomic β-actin was detected in all samples.

### 3.3 Study 2: induction of immune responses by MCF vaccine containing inactivated virus

**Fig. 3:**
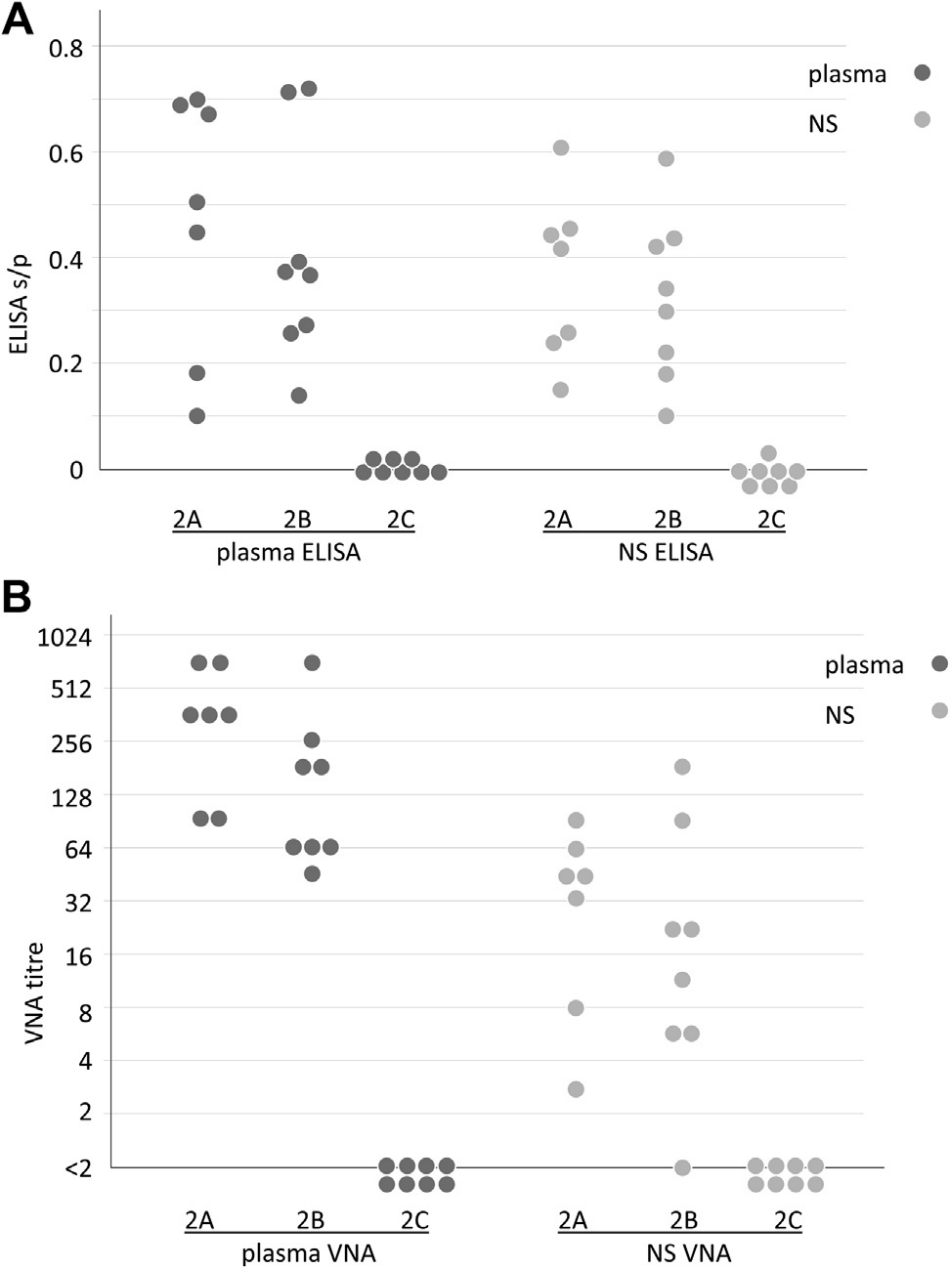
Study 2; ELISA and VNA data. In each panel, the respective values for each animal were plotted by sample type and immunisation group as follows: group 2A, viable AlHV-1 vaccine (mixed with 20% Emulsigen on the day of immunisation); group 2B, inactivated AlHV-1 vaccine (mixed with 20% Emulsigen 28 days before immunisation); group 2C, medium plus 20% Emulsigen adjuvant control. Samples with identical/overlapping values in the same group were plotted offset to each other. Values from d0 samples were essentially zero or undetectable in all samples and therefore were not included. **(A)** d56 ELISA s/p values in blood plasma (plasma ELISA, dark grey circles) and nasal secretions (NS ELISA, light grey circles); **(B)** d56 VNA titres in blood plasma (plasma VNA; dark grey circles) and nasal secretions (NS VNA; light grey circles), plotted on a log2 scale.

Having observed that the attenuated AlHV-1 with adjuvant induced higher neutralising antibody responses compared to attenuated AlHV-1 alone, and that storage of the vaccine virus in the presence of Emulsigen led to >99.9% loss of viability after 1 month, we investigated whether virus viability was important for the induction of virus neutralising antibodies. The results of ELISA and VNA on samples from d0 and d56 are summarised in Fig. 3 and presented in Supplementary Data, Table 3. These show that in the presence of adjuvant both the viable (group 2A) and inactivated (group 2B) formulations of the MCF vaccine induced virus-specific and virus neutralising antibodies in the cattle at d56 (four weeks after second immunisation). Group 2A plasma had median s/p 0.51 (range 0.10–0.70) and median VNA titre 360 (range 90–720); while NS had median s/p 0.42 (range 0.16–0.61) and median VNA titre 45 (range 3–90). One animal in group A was withdrawn from the study before the second immunisation for welfare reasons. Group 2B plasma had median s/p 0.38 (range 0.14–0.72) and median VNA titre 122 (range 45–720); while NS had median s/p 0.32 (range 0.10–0.59) and median VNA titre 17 (range <2–180) (Fig. 3). The antibody responses in the viable (2A) and inactivated (2B) vaccine groups, analysed by *T*-Tests of the s/p values and log2 transformed VNA titres, showed no significant difference between the responses of groups 2A and 2B. Analysis of the untransformed data by non-parametric Mann-Whitney *U* test gave the same outcome.

In contrast, the culture medium plus adjuvant control group (2C) showed no AlHV-1 specific response (plasma and NS s/p values < 0.06; VNA titres < 2 in all samples). Groups 2A and 2B both showed significantly higher virus-specific and virus-neutralising antibody responses at d56, when compared with group 2C, in both VNA and ELISA with p < 0.005 in T-tests of plasma and NS antibodies.

As in study 1, real-time PCR analysis showed that no AlHV-1 DNA was detected in blood samples from study 2 animals at any time point, while genomic β-actin was detected in all samples.

## 4 Discussion

The current vaccine strategy for use of the live attenuated MCF vaccine involves the transport of viable virus and Emulsigen adjuvant (with cold chain) to the study site; the mixing of aliquots of vaccine virus with the appropriate volume of Emulsigen to make a 20% (v/v) final emulsion; and the administration of the mixed vaccine to cattle without undue delay. While this approach has been used successfully in multiple trials in field locations [4], [20], [26], it does not represent an ideal method for deployment of a commercial vaccine. Understanding the factors involved in maintaining the efficacy of this vaccine while simplifying its deployment is therefore of great importance. As a live attenuated vaccine formulated with adjuvant, the current MCF vaccine is unusual. Few commercial live vaccines require adjuvant but examples of live-in-oil vaccines have been reported for Newcastle Disease [28], [29], [30]. To improve our understanding of the MCF vaccine, we have analysed the viability of attenuated AlHV-1, when stored in the presence or absence of adjuvant, and performed immunisation studies focused on two areas: the requirement for adjuvant and the requirement for viable AlHV-1 in the vaccine formulation.

Initial work showed that the viability of AlHV-1 stored in the presence of the oil-in-water adjuvant Emulsigen was progressively reduced at either 4 °C or 20 °C, such that more than 99.9% viability was lost after 1 month and no viable virus could be detected after 2 months. The oil-in-water adjuvant Emulsigen could have reduced virus viability by extraction of the viral envelope and associated membrane proteins into the oil phase. The progressive nature of the loss of viability observed in the presence of Emulsigen (Fig. 1) suggests that this process is slow under the conditions used – storage at the specified temperature with daily mixing. Additionally, it was observed that the vaccine aliquots separated into aqueous and emulsion phases if left undisturbed, suggesting that loss of viability might be accelerated by continuous mixing.

In study 1, the virus-specific antibody data (ELISA) suggested that immunisation with viable AlHV-1 in the absence of adjuvant (group 1B) was associated with a change in the dynamics of plasma antibody responses (Fig. 2A) and significantly lower nasal secretion antibody responses (Fig. 2B) after the second immunisation. In addition, the results showed that group 1B animals had significantly lower plasma and NS VNA titres at day 69 post-immunisation than group 1A animals, which received the AlHV-1 vaccine containing Emulsigen. These observations suggest that although attenuated viable AlHV-1 can induce a virus-neutralising immune response in the absence of adjuvant, the magnitude of this antibody response is significantly enhanced by formulation with adjuvant. This is consistent with studies of Newcastle Disease Virus vaccines, whose immunogenicity could be potentiated by formulation with oil adjuvants [30], [29]. Thus, there may be a qualitative and quantitative difference in the immune responses induced in the presence or absence of adjuvant, with the virus-only immunisation leading to a reduced virus neutralising antibody response that may be less protective. The presence of adjuvant may lead to a depot effect, concentrating the virus near the site of injection and facilitating its slow release and prolonged delivery to local lymph nodes by antigen-presenting cells. In the absence of adjuvant, the virus may be distributed rapidly via the circulation and cleared by immune cells resulting is a less prolonged stimulation. A reduced local and/or reduced prolonged response in the absence of adjuvant may explain the reduction in both virus-specific and virus-neutralising antibodies in nasal secretion samples of group 1B. The apparent lack of replication of attenuated AlHV-1 in vaccinated cattle may explain its inability to induce high levels of neutralising antibodies and generate an effective anamnestic response in the absence of the depot effect conferred by adjuvant.

In study 2, we compared the AlHV-1 vaccine containing Emulsigen, prepared and used while virus viability was high, with the same vaccine formulation after storage had reduced virus viability by >99.9%. The antibody responses in the respective groups at d56 showed that both MCF vaccine formulations induced virus-specific and virus-neutralising antibody responses of similar magnitude, while the control group had no virus-specific response (Fig. 3). This supports the view that viable AlHV-1 is not required for induction of optimal virus-specific or virus neutralising antibody responses following vaccination when combined with Emulsigen. The induction of a reduced virus neutralising immune response following immunisation by attenuated AlHV-1 without adjuvant, and the lack of detection of AlHV-1 in the blood samples taken during both studies, support the view that the vaccine acts as a source of antigen and that significant virus replication or persistence of viable virus is not required for the development of neutralising antibody responses in the bovine host.

Studies of potential MCF vaccines based on cell-culture-derived AlHV-1 were reported in 1975 and 1980 [22], [31]. These publications described the use of AlHV-1 strains WC11 (non-pathogenic) or C500 (pathogenic) in live or inactivated vaccines inoculated into cattle or rabbits with Freund’s complete or incomplete adjuvants. In each case, circulating virus-neutralising antibody responses were detected over several months in vaccinated cattle, with titres in excess of 100. However, protection from MCF, using intravenous challenge or field exposure to virus, was not demonstrated in cattle. We previously observed that the attenuated MCF virus vaccine induced protection against a challenge by intranasal inoculation of cell-free pathogenic AlHV-1, whilst intravenous challenge was fatal [19]. The induction of virus neutralising antibodies by earlier vaccine formulations was demonstrated only within blood samples so it cannot be determined whether mucosal antibody responses had been induced. However, these studies suggest that chemically-inactivated AlHV-1, with appropriate adjuvants, might also induce protective mucosal virus-neutralising antibody responses.

We have shown that protection from MCF may be associated with the induction of virus-neutralising antibodies in the mucous secretions of immunised animals [19], [20], [21], [25]. The results described here, that vaccination with attenuated AlHV-1 without adjuvant elicited a significantly lower titre of virus-neutralising antibodies than the equivalent adjuvanted vaccine, and that MCF vaccine containing inactivated AlHV-1 induced similar antibody responses to the vaccine containing viable virus, suggest that while the current formulation of the MCF vaccine mixes viable virus suspension with Emulsigen adjuvant just before use, other formulations containing adjuvant and non-viable AlHV-1 may also elicit a protective response.

This speculation agrees with previous work [32], in which vaccination of rabbits with DNA encoding the OvHV-2 gB, gH and gL glycoproteins induced virus neutralising antibodies and appeared to protect the rabbits from intranasal challenge with OvHV-2, demonstrating protection by appropriately presented viral antigens. However, it will be important to investigate the efficacy of vaccine formulations that contain inactivated AlHV-1 or which lack adjuvant. Indeed, the poor mucosal response seen in the virus-only group of study 1 (group 1B, Fig. 2B) might be improved by mucosal inoculation of attenuated AlHV-1. Previous work, boosting by the intranasal route in combination with mucosal adjuvants, induced moderate titres of virus-neutralising antibodies and demonstrated some protection from challenge, while VNA titres following vaccination with formalin-inactivated virus were low [19], suggesting that these areas require further research. This work therefore represents progress towards the development of a commercial vaccine for WA-MCF, especially if both virus inactivation and freeze-drying do not reduce efficacy, and may help facilitate the distribution of vaccine to at-risk cattle herds by removing the need for an extensive cold-chain.

## 5 Animal welfare

All animal experiments were performed with the approval of the Moredun Research Institute’s Animal Welfare and Ethical Review Body (AWERB) in compliance with the UK “Animals (Scientific Procedures) Act 1986”, with a maximum severity limit of mild.

## CRediT authorship contribution statement

**George C. Russell:** Conceptualization, Formal analysis, Data curation, Funding acquisition, Investigation, Methodology, Project administration, Resources, Supervision, Validation, Visualization, Writing - original draft, Writing - review & editing. **David M. Haig:** Conceptualization, Funding acquisition, Resources, Supervision, Writing - original draft, Writing - review & editing. **Mark P. Dagleish:** Conceptualization, Formal analysis, Investigation, Resources, Supervision, Writing - review & editing. **Helen Todd:** Conceptualization, Investigation, Formal analysis, Data curation, Methodology, Writing - review & editing. **Ann Percival:** Conceptualization, Investigation, Formal analysis, Data curation, Methodology, Writing - original draft, Writing - review & editing. **Dawn M. Grant:** Conceptualization, Investigation, Methodology, Supervision, Validation, Writing - original draft, Writing - review & editing. **Jackie Thomson:** Conceptualization, Investigation, Methodology, Supervision, Writing - review & editing. **Anna E. Karagianni:** Conceptualization, Formal analysis, Data curation, Investigation, Methodology, Validation, Visualization, Writing - review & editing. **Julio Benavides:** Conceptualization, Formal analysis, Data curation, Investigation, Methodology, Supervision, Visualization, Writing - original draft, Writing - review & editing.

## Declaration of Competing Interest

The authors declare that they have no known competing financial interests or personal relationships that could have appeared to influence the work reported in this paper.
