# Supplementary material for: Analysis of immune responses to attenuated alcelaphine herpesvirus 1 formulated with and without adjuvant
Source: Vaccine X. 2021 Mar 22;8:100090. doi: 10.1016/j.jvacx.2021.100090 (PMC8065228; doi:10.1016/j.jvacx.2021.100090)
Supplement: Supplementary Data 1 [file mmc1.pdf]

Supplementary data

Supplementary Table 1. Analysis of virus viability in the presence of 20% Emulsigen – virus used in study 2

	4 °C		20 °C	
day	virus alone	virus Emulsigen	virus alone	virus Emulsigen
7	$2.0x10^9$	$6.3x10^5$	$2.0x10^8$	$6.3x10^5$
14	$1.1x10^9$	$6.3x10^4$	$6.3x10^7$	$1.1x10^4$
28	$1.1x10^8$	$2.0x10^3$	$6.3x10^4$	$6.3x10^2$
63	$2.0x10^7$	$<10^2$*	$3.5x10^5$	$< 10^2$*

* Limit of detection of the assay system based on lack of virus detection at the lowest dilution of 10^{-1}.

Supplementary Table 2. Study 1 serological results.

For each animal, ELISA S/P before vaccination (d0), and approximately fortnightly thereafter, was measured in samples of blood plasma and nasal secretion (NS). ELISA data are expressed as sample to positive (S/P) ratio, where the positive sample for the assays was a pool of plasma or NS from previously vaccinated and tested cattle (Russell et al., 2012). VNA titres were measured before vaccination (d0) and at the end of the trial (d69) to ensure immune responses in both plasma and NS antibody responses were likely to be protective. VNA titres expressed as <2 indicate that no neutralisation was detected at the lowest dilution that could be used (1:2 final).

Groups	Virus Neutralisation Assays*					ELISA values												
Animals	Plasma titre		NS titre			Plasma ELISA S/P							NS ELISA S/P					
	d0	d69	d0	d69		d0	d14	d28	d47	d56	d69		d0	d14	d28	d47	d56	d69
**Group 1A**																		
194	<2	720	<2	90		0.000	0.096	0.061	0.803	1.096	0.711		0.025	0.000	0.000	0.578	0.384	0.000
692	3	2048	<2	45		0.000	0.000	0.000	0.624	0.552	0.230		0.001	0.047	0.000	0.646	0.528	0.329
573	<2	512	<2	32		0.000	0.000	0.000	0.218	0.093	0.157		0.000	0.000	0.000	0.666	0.616	0.399
423	4	360	<2	64		0.000	0.109	0.185	1.081	0.598	0.418		0.000	0.067	0.000	0.299	0.425	0.236
197	3	512	<2	32		0.031	0.143	0.176	0.509	0.654	0.504		0.000	0.000	0.000	0.193	0.067	0.132
580	<2	360	<2	11		0.044	0.036	0.034	0.446	0.403	0.384		0.000	0.000	0.000	0.127	0.104	0.086
**Group 1B**																		
291	3	90	<2	6		0.152	0.121	0.310	0.290	0.431	0.402		0.000	0.000	0.000	0.071	0.019	0.000
777	<2	90	<2	6		0.000	0.151	0.205	0.275	0.257	0.447		0.000	0.000	0.000	0.000	0.000	0.000
420	11	45	<2	<2		0.138	0.147	0.000	0.000	0.000	0.000		0.000	0.076	0.042	0.050	0.000	0.017
772	<2	90	<2	6		0.414	1.691	0.441	0.706	0.408	0.524		0.000	0.267	0.082	0.099	0.017	0.134
129	<2	45	<2	8		0.000	0.491	0.000	0.003	0.230	0.098		0.061	0.050	0.033	0.030	0.000	0.000
426	4	32	<2	<2		0.275	0.315	0.319	0.489	0.304	0.190		0.021	0.000	0.000	0.055	0.094	0.027
**Group 1C**																		
195	<2	<2	<2	<2		0.000	0.000	0.182	0.000	0.053	0.000		0.000	0.000	0.001	0.010	0.001	0.019
690	<2	<2	<2	<2		0.034	0.279	0.000	0.267	0.334	0.000		0.000	0.000	0.000	0.000	0.000	0.000
811	<2	<2	<2	<2		0.000	0.000	0.029	0.129	0.000	0.000		0.000	0.000	0.000	0.000	0.000	0.000

183	<2	<2	<2	<2		0.183	0.043	0.000	0.073	0.000	0.000		0.000	0.027	0.006	0.076	0.000	0.022
581	<2	<2	<2	<2		0.000	0.000	0.000	0.000	0.000	0.058		0.001	0.000	0.000	0.000	0.000	0.000
199	<2	<2	<2	3		0.000	0.000	0.000	0.000	0.000	0.044		0.000	0.000	0.000	0.008	0.000	0.000
064	<2	<2	<2	<2		0.000	0.058	0.000	0.000	0.083	0.000		0.000	0.000	0.000	0.022	0.000	0.000

Supplementary Table 3. Study 2 serological results.

Blood plasma and nasal secretion (NS) samples taken before first immunisation (d0) and following the peak of immune response (d56) were tested in virus neutralisation assays (VNA) and ELISA for AlHV-1 specific antibodies. VNA titres expressed as <2 indicate that no neutralisation was detected at the lowest dilution that could be used. ELISA data are expressed as sample to positive (S/P) ratio, where the positive sample for the assays was a pool of plasma or NS from previously vaccinated and tested cattle (Russell et al., 2012).

**Groups**	**Virus Neutralisation Assays**						**ELISA**				
**Animals**	**Plasma titre**			**NS titre**			**Plasma ELISA S/P**			**NS ELISA S/P**	
	**d0**	**d56**		**d0**	**d56**		**d0**	**d56**		**d0**	**d56**
**Group 2A**											
**409**	<2	360		<2	8		0.01	0.45		0.00	0.24
**406**	3	360		<2	45		0.00	0.19		0.00	0.42
**258**	<2	360		<2	90		0.00	0.51		0.06	0.26
**043**	<2	90		<2	3		0.00	0.10		0.00	0.16
**049**	<2	720		<2	45		0.02	0.70		0.00	0.44
**054**	<2	90		<2	32		0.00	0.69		0.00	0.46
**206**	<2	720		<2	64		0.01	0.68		0.00	0.61
**Group 2B**											
**410**	<2	180		<2	90		0.01	0.37		0.00	0.42
**413**	<2	64		<2	11		0.01	0.26		0.00	0.30
**041**	<2	64		<2	<2		0.00	0.14		0.00	0.10
**044**	<2	45		<2	22		0.01	0.39		0.00	0.34
**051**	<2	64		<2	6		0.00	0.28		0.00	0.18
**058**	<2	256		<2	22		0.00	0.72		0.00	0.43
**040**	<2	180		<2	6		0.01	0.38		0.00	0.22
**203**	<2	720		<2	180		0.00	0.71		0.00	0.59
**Group 2C**											

**412**	<2	<2		<2	<2		0.01	0.00		0.00	0.00
**414**	<2	<2		<2	<2		0.06	0.00		0.00	0.00
**042**	<2	<2		<2	<2		0.00	0.00		0.00	0.00
**045**	<2	<2		<2	<2		0.03	0.01		0.00	0.00
**053**	<2	<2		<2	<2		0.01	0.00		0.00	0.00
**059**	<2	<2		<2	<2		0.01	0.01		0.00	0.01
**217**	<2	<2		<2	<2		0.00	0.00		0.00	0.00
**698**	<2	<2		<2	<2		0.01	0.01		0.00	0.00
